# A Rare Case of Exophiala Dermatitidis Isolation in a Patient with Non-Cystic Fibrosis Bronchiectasis: Colonization or True Infection?

**DOI:** 10.3390/diagnostics15131661

**Published:** 2025-06-29

**Authors:** Francesco Rocco Bertuccio, Nicola Baio, Simone Montini, Valentina Ferroni, Vittorio Chino, Lucrezia Pisanu, Marianna Russo, Ilaria Giana, Elisabetta Gallo, Lorenzo Arlando, Klodjana Mucaj, Mitela Tafa, Maria Arminio, Emanuela De Stefano, Alessandro Cascina, Angelo Guido Corsico, Giulia Maria Stella, Valentina Conio

**Affiliations:** 1 Unit of Respiratory Disease, Cardiothoracic and Vascular Department, IRCCS Policlinico San Matteo, Viale Golgi 19, 27100 Pavia, Italy; f.bertuccio@smatteo.pv.it (F.R.B.); simone.montini01@universitadipavia.it (S.M.); valentina.ferroni01@universitadipavia.it (V.F.); lucrezia.pisanu01@universitadipavia.it (L.P.); marianna.russo01@universitadipavia.it (M.R.); ilaria.giana01@universitadipavia.it (I.G.); elisabetta.gallo01@universitadipavia.it (E.G.); lorenzo.arlando01@universitadipavia.it (L.A.); klodjana.mucaj01@universitadipavia.it (K.M.); mitela.tafa01@universitadipavia.it (M.T.); maria.arminio01@universitadipavia.it (M.A.); emanuela.destefano01@universitadipavia.it (E.D.S.); a.cascina@smatteo.pv.it (A.C.); a.corsico@smatteo.pv.it (A.G.C.); 2 Department of Internal and dicine and Pharmacology, University of Pavia, 27100 Pavia, Italy; 3 Ospedale Maggiore, ASST Crema, 26013 Crema, Italy; nicola.baio01@universitadipavia.it; 4 Ospedale Pederzoli, Peschiera del Garda, 37121 Verona, Italy; vittorio.chino01@universitadipavia.it

**Keywords:** *Exophiala dermatitidis*, fungal lung infection, dematiaceous fungi, colonization, non-cystic bronchiectasis, chronic airway disease, voriconazole, immunocompetent host

## Abstract

**Background:** *Exophiala dermatitidis* is a dematiaceous, thermotolerant, yeast-like fungus increasingly recognized as an opportunistic pathogen in chronic airway diseases. While commonly associated with cystic fibrosis, its clinical significance in non-cystic fibrosis bronchiectasis (NCFB) remains unclear. **Case Presentation:** We report the case of a 66-year-old immunocompetent woman with a history of breast cancer in remission and NCFB, who presented with chronic cough and dyspnea. Chest CT revealed bilateral bronchiectasis with new pseudonodular opacities. Bronchoalveolar lavage cultures identified *E. dermatitidis*, along with *Pseudomonas aeruginosa* and methicillin-sensitive *Staphylococcus aureus*. Given clinical stability and the absence of systemic signs, initial therapy included oral voriconazole, levofloxacin, doxycycline, and inhaled amikacin. Despite persistent fungal isolation on repeat bronchoscopy, the patient remained asymptomatic with stable radiologic and functional findings. Antifungal therapy was discontinued, and the patient continued under close monitoring. The patient exhibited clinical and radiological stability despite repeated fungal isolation, reinforcing the hypothesis of persistent colonization rather than active infection. **Discussion:** This case underscores the diagnostic challenges in distinguishing fungal colonization from true infection in structurally abnormal lungs. In NCFB, disrupted mucociliary clearance and microbial dysbiosis may facilitate fungal persistence, even in the absence of overt immunosuppression. The detection of *E. dermatitidis* should prompt a comprehensive evaluation, integrating clinical, radiologic, and microbiologic data to guide management. Voriconazole is currently the antifungal agent of choice, though therapeutic thresholds and duration remain undefined. **Conclusions:** This report highlights the potential role of *E. dermatitidis* as an under-recognized respiratory pathogen in NCFB and the importance of a multidisciplinary, individualized approach to diagnosis and treatment. This case underscores the need for further research on fungal colonization in NCFB and the development of evidence-based treatment guidelines. Further studies are needed to clarify the pathogenic significance, optimal management, and long-term outcomes of *E. dermatitidis* in non-CF chronic lung diseases.

## 1. Introduction

*Exophiala dermatitidis* is a dematiaceous, thermotolerant fungus increasingly recognized for its opportunistic potential in the human respiratory tract. Though traditionally viewed as an environmental saprophyte, it has emerged as a pathogen of interest, particularly in patients with chronic pulmonary diseases such as cystic fibrosis (CF), where its prevalence may reach up to 19% in respiratory samples. Despite this, its clinical significance outside of CF remains poorly understood, with very few cases reported in non-cystic fibrosis bronchiectasis (NCFB) or structurally abnormal lungs [1,2,3].

NCFB is a chronic airway disorder characterized by irreversible bronchial dilatation, impaired mucociliary clearance, and persistent microbial colonization. These factors create a permissive niche not only for typical bacterial pathogens such as *Pseudomonas aeruginosa* and *Staphylococcus aureus* but also for less common fungal organisms. Repeated antibiotic exposure, mucus retention, and local immune dysregulation further disrupt the airway microbiome, potentially facilitating fungal persistence and pathogenesis [4,5,6].

In this context, the isolation of *E. dermatitidis* raises a critical diagnostic dilemma: does it represent mere colonization or an active infection warranting antifungal treatment? This distinction is essential, as treatment protocols are not standardized, susceptibility testing is limited, and prolonged azole therapy may carry significant toxicity. Voriconazole is generally considered the first-line agent due to its antifungal spectrum and good pulmonary bioavailability, although the duration and indications for therapy remain debated and largely based on expert opinion or case series [7,8,9].

Here, we report a rare case of *E. dermatitidis* isolated from the bronchoalveolar lavage of an immunocompetent woman with NCFB alongside *P. aeruginosa* and *S. aureus* co-colonization. The case underscores the importance of integrating clinical, radiologic, and microbiologic data to guide individualized decision-making, and it contributes to the growing body of literature on emerging fungal pathogens in non-CF airway diseases. To date, only a limited number of cases of *Exophiala dermatitidis* pulmonary isolation have been reported in patients with non-cystic fibrosis bronchiectasis, especially among immunocompetent individuals. This report contributes to the growing but still scarce body of literature on the clinical implications of rare fungal colonization in structurally abnormal lungs.

## 2. Case Presentation

A 66-year-old woman presented to the respiratory disease outpatient clinic with a prolonged history of persistent cough and shortness of breath during exertion. Her symptoms had gradually developed over the past months. She denied sputum production, wheezing, night sweats, weight loss, or fever. Her history was notable for a remote history of cigarette smoking (10 pack-years), having quit five years earlier. She denied any known exposure to birds, hot tubs, or mold and reported no known allergies. The patient was a retired office employee, without recent travel or relevant occupational/environmental exposures. She was not taking chronic medications at the time of presentation.

The patient had a significant oncologic history. Nine years ago, she was diagnosed with left-sided breast cancer and underwent neoadjuvant chemotherapy followed by a left radical mastectomy. Five years later, she experienced a local recurrence and began another chemotherapy treatment with docetaxel, trastuzumab, and pertuzumab. Restaging PET showed no evidence of distant metastases or recurrence. As of the most recent follow-up, her oncologic disease remains in remission.

Three years earlier, she had begun experiencing similar respiratory symptoms, and a workup at that time revealed bronchiectasis with mucoid impactions and multiple tiny pulmonary nodules. Bronchoalveolar lavage (BAL) cultures identified *Mycobacterium chimaera*, sensitive to amikacin, clarithromycin, and linezolid. A 12-month course of targeted therapy led to the eradication of the mycobacterial infection.

### 2.1. Physical Examination Findings

At presentation, the patient appeared in no acute distress. Her vital signs were stable: no fever, heart rate 78 bpm, normal blood pressure and respiratory rate, and oxygen saturation of 97% on room air. Lung auscultation revealed fine bilateral inspiratory crackles, without wheezes or rhonchi. No digital clubbing or peripheral edema was observed. Cardiac, abdominal, and neurologic examinations were unremarkable.

### 2.2. Diagnostic Studies

Initial blood tests, including complete blood count, liver and kidney function tests, electrolytes, and glucose, were all within normal limits. Inflammatory markers were not elevated (C-reactive protein, erythrocyte sedimentation rate, and procalcitonin were within normal ranges). The serologic workup for connective tissue diseases was negative, including ANA, ANCA, and anti-SSA/SSB antibodies. HIV and viral hepatitis screening were negative. Immunoglobulin levels (IgA, IgE, IgG, IgM) and alpha-1 antitrypsin were within the normal range. Serum β-D-glucan was not performed, while serum galactomannan was borderline (0.19), not consistent with invasive fungal infection.

Pulmonary function tests showed normal static and dynamic lung volumes with a preserved diffusing capacity for carbon monoxide: FVC 90% of predicted; RV 102% of predicted; TLC 95 of predicted %; FEV1 93% of predicted; DLCO 83% of predicted.

**Figure 1:**
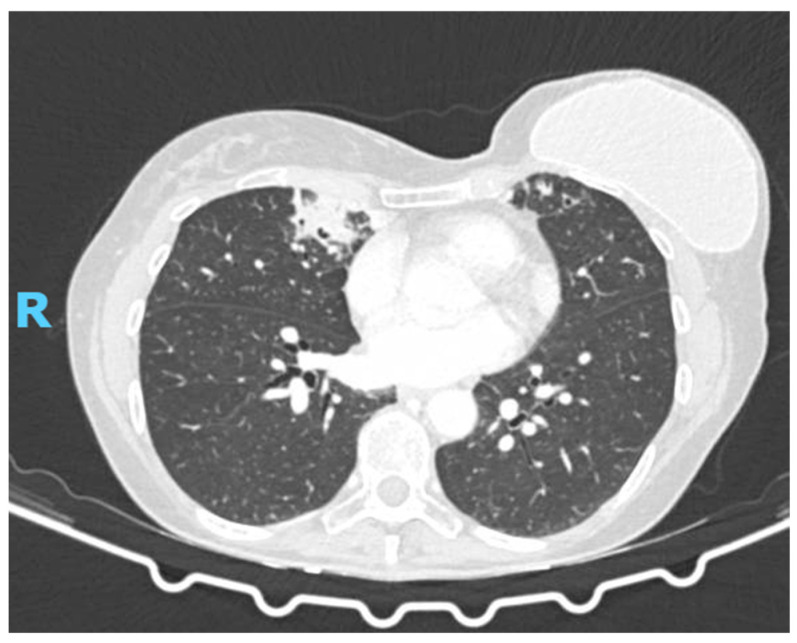
The chest CT scan revealed new pseudonodular opacities in the middle lobe.

Repeat high-resolution chest CT (Figure 1) revealed new pseudonodular opacities in the middle lobe and left lower lobe, randomly distributed with an inflammatory appearance, without signs of neoplastic recurrence. Background findings of bilateral bronchiectasis, mucus impaction, and bronchial wall thickening were unchanged. Bronchoscopy with bronchoalveolar lavage was executed.

### 2.3. What Is the Diagnosis?

The diagnosis was pulmonary infection by Exophiala dermatitidis in a patient with non-cystic fibrosis bronchiectasis.

## 3. Discussion

*Exophiala dermatitidis* is a dematiaceous (black) yeast-like fungus ubiquitously found in the environment, including decaying organic matter and moist indoor surfaces. Belonging to the *Herpotrichiellaceae* family, it exhibits thermotolerance and thrives in humid habitats. Though occasionally isolated from respiratory specimens, clinically relevant pulmonary infections by *Exophiala* species are rare, particularly among immunocompetent hosts [7,10,11,12].

Invasive and disseminated infections are mostly reported in immunocompromised patients or those with hematologic malignancies, organ or stem cell transplants, or prolonged neutropenia [7,13]. However, an increasing number of case reports have identified *E. dermatitidis* as an emerging opportunistic pathogen in patients with chronic respiratory diseases, particularly cystic fibrosis (CF) and, more recently, bronchiectasis [9]. In non-CF bronchiectasis, the organism’s role remains poorly defined, with colonization versus infection often challenging to distinguish.

Predisposing factors such as impaired mucociliary clearance, structural airway damage, mucus retention, and frequent antibiotic exposure likely facilitate colonization and, in certain cases, progression to infection. While the presence of *E. dermatitidis* in sputum may represent harmless colonization in some, it can trigger an inflammatory response, exacerbate respiratory symptoms, or evolve into invasive disease in others [14,15].

Bronchiectasis is characterized by permanent bronchial dilation, disrupted mucociliary function, and chronic mucus retention. This creates a nutrient-rich, hypoxic microenvironment conducive to microbial persistence [4]. Dematiaceous fungi such as *Exophiala*, with biofilm-forming capacity and thermotolerance, can exploit this niche. Additionally, repeated use of broad-spectrum antibiotics disrupts the microbial balance of the airway, potentially promoting fungal overgrowth and dysbiosis [16].

Notably, interactions between *E. dermatitidis* and common bacterial pathogens may influence fungal behavior and host immune responses. *P. aeruginosa* is known to secrete quorum-sensing molecules and iron-chelating agents that can inhibit or modulate fungal growth, while *S. aureus* may enhance fungal persistence through biofilm co-aggregation and immune evasion. These interactions may shift the microbial community toward chronic inflammation or facilitate transitions from colonization to infection. In particular, coinfection or sequential colonization with these bacteria might predispose the lung to fungal persistence or pathogenesis [16,17].

Although often asymptomatic, fungal colonization may elicit an aberrant immune response in certain patients, manifesting as increased cough, sputum production, radiologic changes, and clinical deterioration. In rare cases, especially with additional risk factors such as prior cancer therapy or subtle immunologic defects, *E. dermatitidis* can invade pulmonary tissue, leading to bronchopneumonia, necrotizing infections, or systemic dissemination [16].

Recognizing this potential transition is critical for timely diagnosis and management. Increasing awareness of such fungal pathogens in non-CF bronchiectasis is essential for guiding future therapeutic strategies and for refining our understanding of host–pathogen–microbiome interactions in chronic airway diseases.

Symptoms are often nonspecific, cough, sputum, and dyspnea, and overlap with baseline bronchiectasis manifestations. Imaging findings are also variable. In our case, chest CT showed bronchiectasis, mucus plugging, and new pseudonodular inflammatory opacities, raising suspicion for fungal involvement [18]. These were not consistent with cancer recurrence or NTM reactivation, both of which had been previously addressed.

The isolation of *E. dermatitidis* from respiratory samples confirms diagnosis, ideally supported by repeat cultures, growth on Sabouraud dextrose agar, and molecular identification. Histopathological confirmation is rare and usually reserved for suspected invasive disease [19,20,21]. Given the overlap with colonization, clinical judgment remains essential in deciding whether to initiate antifungal therapy.

Treatment remains non-standardized due to the rarity of cases. In vitro susceptibility profiles show variable responses, but azoles, particularly voriconazole and posaconazole, demonstrate consistent activity. Amphotericin B shows less efficacy and is usually reserved for severe infections or as induction therapy. Voriconazole is the most commonly used agent, likely due to its excellent lung penetration and activity against melanized fungi. It is employed as monotherapy or following initial induction with amphotericin B or itraconazole. Itraconazole is often used orally in milder cases or as a step-down agent. Posaconazole is generally reserved for cases with azole intolerance or resistance [9,18,22,23,24,25,26,27,28,29,30,31].

Although specific treatment durations are seldom reported, available evidence suggests regimens typically last 3 to 6 months, with prolonged therapy (>6 months) in patients with structural lung disease or coinfections (e.g., NTM). In a few cases, surgical resection was performed, either due to localized disease or inadequate response to medical therapy, highlighting the potential utility of a combined medical–surgical approach in selected patients [9,18,22,23,24,25,26,27,28,29,30,31].

Clinical outcomes are generally favorable, particularly in immunocompetent individuals. The only fatality in the reviewed literature involved a patient with multiple comorbidities and likely immunosuppression. Thus, individualized therapeutic strategies based on clinical severity, host status, radiologic progression, and microbiological findings are essential [32,33,34,35].

In our case, the patient received a 12-week course of oral voriconazole, combined with levofloxacin and doxycycline targeting concurrent bacterial pathogens. Inhaled amikacin was added to address chronic *Pseudomonas* colonization. Voriconazole was initiated despite the absence of systemic symptoms, given the presence of new inflammatory opacities on imaging, the co-isolation of multiple pathogens, and the patient’s prior history of opportunistic infection (NTM infection).

Follow-up imaging showed stable disease, and repeat BAL continued to yield *E. dermatitidis* without clinical worsening. Given the absence of deterioration, further antifungal therapy was withheld in agreement with infectious disease specialists, suggesting a colonization phenotype rather than active infection.

This case is of particular scientific and clinical relevance due to the rarity of *E. dermatitidis* infection in immunocompetent patients with non-CF bronchiectasis. While its role as an opportunistic pathogen is increasingly recognized, most cases involve individuals with CF, hematologic malignancy, or immunosuppression. Reports in structurally abnormal but immunocompetent lungs remain scarce. In our case, it could be plausible that the patient’s prior exposure to systemic chemotherapy and monoclonal antibodies may have caused long-lasting subclinical immune dysregulation, thereby promoting fungal persistence. In Table 1, we describe briefly the main case reports published in the literature despite the fact that they are rare. Describing such cases contributes to expanding our understanding of the potential role of dematiaceous fungi in chronic respiratory conditions and highlights the diagnostic and therapeutic uncertainties they present.

Moreover, this report underscores the need for heightened awareness of atypical fungal pathogens in patients with bronchiectasis, especially when standard therapies fail or when radiologic findings are unusual. Reporting rare but well-characterized infections enhances evidence-based decision-making, reveals emerging susceptibility patterns, and helps define the natural history of fungal colonization in chronic airway disease.

Rather than merely presenting an unusual isolation, our aim is to stimulate broader interest in under-recognized fungal pathogens, emphasize the complexity of host–microbe interactions in bronchiectasis, and support future research aimed at developing tailored diagnostic tools and antifungal treatment protocols. Ultimately, this case highlights the critical role of multidisciplinary collaboration, spanning pulmonology, infectious diseases, microbiology, and radiology, in managing complex airway infections and improving patient outcomes.

### Clinical Course

**Figure 2:**
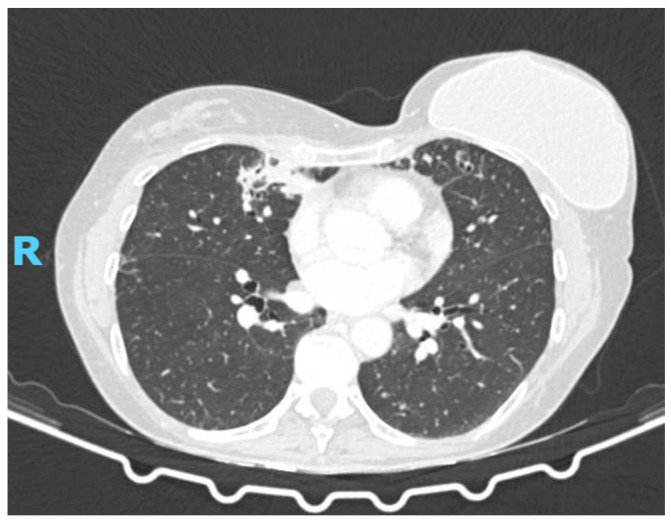

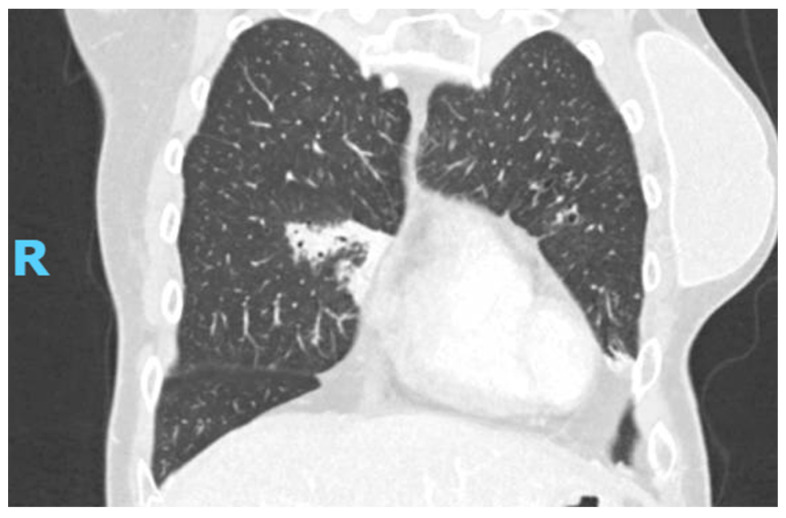
Follow-up chest CT scan revealed persistent inflammatory opacities without new consolidations.

The patient’s bronchoscopy revealed isolation of Exophiala dermatitidis, along with Pseudomonas aeruginosa and methicillin-sensitive Staphylococcus aureus. She was initially treated with oral levofloxacin for 14 days, doxycycline for 7 days, and aerosolized amikacin for 15 days. A 12-week course of voriconazole (200 mg twice per day) was also initiated. The follow-up chest CT scan revealed persistent inflammatory opacities without new consolidations (Figure 2). Repeat BAL again grew Exophiala dermatitidis, and galactomannan was borderline (0.19) without other bacterial isolations. Given clinical stability and the absence of new respiratory symptoms, further antifungal therapy was withheld. At her most recent evaluation, she remained asymptomatic, with stable pulmonary function and imaging.

## 4. Conclusions

This case highlights the emerging relevance of *Exophiala dermatitidis* as a potential respiratory pathogen in patients with chronic lung disease, even in the absence of overt immunosuppression. Although rare, *E. dermatitidis* should be considered in the differential diagnosis of new pulmonary infiltrates, particularly in individuals with structural airway abnormalities such as non-cystic fibrosis bronchiectasis (NCFB).

The distinction between colonization and active infection remains a significant clinical challenge. As shown in this case, a comprehensive, longitudinal evaluation incorporating clinical symptoms, radiological evolution, and repeated microbiological findings is often necessary to guide therapeutic decisions and avoid both under- and overtreatment.

When antifungal therapy is deemed appropriate, voriconazole currently represents the first-line agent due to its demonstrated efficacy and favorable pharmacokinetics in pulmonary tissue. However, standardized treatment protocols are lacking, and the optimal duration of therapy remains uncertain, requiring individualized assessment based on disease severity, response to treatment, and host factors.

Importantly, NCFB itself constitutes a predisposing condition for unusual infections, as impaired mucociliary clearance, mucus stasis, and frequent antibiotic exposure create a permissive environment for opportunistic fungal colonization and, potentially, pathogenic invasion—even in immunocompetent patients.

This report is limited by the absence of histopathological confirmation and the relatively short duration of follow-up. Moreover, the lack of standardized guidelines for managing fungal infections in non-CF bronchiectasis represents a significant challenge in clinical decision-making.

This case underscores the value of a multidisciplinary approach encompassing pulmonology, infectious diseases, microbiology, and radiology in the management of rare pulmonary fungal infections. Collaboration among specialties not only facilitates timely diagnosis and tailored treatment but also contributes to the growing body of knowledge required to better characterize the clinical significance and natural history of under-recognized fungal pathogens in chronic airway diseases. Future studies should explore host immune mechanisms and microbiome interactions that may underlie chronic fungal colonization in patients with bronchiectasis.

## Figures and Tables

**Table 1:** Main case reports published.

Author (Reference)	Age	Sex	Country	Underlying Disease(s)	Symptoms	Treatment	Antifungal Therapy and Estimated Duration	Notes
Barenfanger et al. [31]	79	F	USA	Bronchiectasis	Hemoptysis, fever	Amphotericin B, 5-FC	Amphotericin B + 5-FC (IV), ~6–8 weeks	Likely IV start, step-down unknown
Kenney et al [36]	21	F	USA	Chronic granulomatous disease	Fever, chills, SOB	Surgery, Amphotericin B, 5-FC, Fluconazole	Combination therapy, ~6–12 weeks + oral Fluconazole	Immunocompromised host
Mukaino et al. [30]	54	F	Japan	Bronchiectasis	Cough, sputum	Miconazole, Amphotericin B	Likely topical/oral Miconazole + IV AmB, ~4–6 weeks	Non-severe
Taj-Aldeen et al. [29]	54	F	Quatar	Diabetes, cervical cancer	Cough, sputum, hemoptysis	Fluconazole, Itraconazole, Amphotericin B	Likely stepwise approach, total ~2–3 months	Fatal outcome, likely severe
Ozawa et al. [28]	81	F	Japan	None	Hemoptysis	Itraconazole	Itraconazole PO, ~3 months	Good response
Tanamachi et al. [37]	53	F	Japan	Bronchiectasis	Sputum, chest pain	Miconazole, 5-FC, Itraconazole	Combination (oral), likely ~3–6 months	Oral triple therapy
Bulloch et al. [27]	86	F	USA	Dementia	Asymptomatic	Voriconazole	VRCZ PO, likely short course (~4 weeks)	Incidental finding
Suzuki et al. [18]	65	M	Japan	Multiple myeloma	Asymptomatic	Voriconazole, Surgery	VRCZ PO, likely ~6 weeks post-surgery	Immunosuppressed
Mukai et al. [26]	63	F	Japan	None	Chest pain	Itraconazole	ITCZ PO, ~3 months	Standard regimen
Shintani et al. [38]	56	F	Japan	Bronchiectasis	Sputum, fever	Itraconazole	ITCZ PO, ~3–4 months	Bronchiectasis context
Goto et al. [39]	70	F	Japan	NTM	Sputum, chest pain	Voriconazole	VRCZ PO, ~4–6 months	NTM co-infection
Masuo et al. [40]	58	F	Japan	NTM	Cough	Voriconazole	VRCZ PO, ~4–6 months	NTM context
Sekiguchi et al. [25]	65	F	Japan	RA, bronchiectasis	Cough, sputum	Voriconazole	VRCZ PO, ~6 months	Autoimmune comorbidity
Li et al. [24]	52	M	China	None	Cough, sputum, hemoptysis	VRCZ, Amphotericin B, Posaconazole	Escalated therapy, total ~3–4 months	Likely initial failure
Watanabe et al. [23]	65	F	Japan	NTM, sinusitis	Cough, sputum	Voriconazole	VRCZ PO, ~3–4 months	Coinfection context
Watanabe et al. [23]	47	F	Japan	RA, NTM, sinusitis	Nasal discharge, anosmia	Amphotericin B, VRCZ, Itraconazole	Sequential therapy, total ~6 months	Upper + lower airway involvement

## Data Availability

The data that support the findings of this study are available on request from the corresponding author.

## References

[1] Bahadori T., Didehdar M., Khansarinezhad B., Shokohi T. (2020). Identification of Opportunistic and Nonopportunistic Exophiala Species Using High Resolution Melting Analysis. Med. Mycol..

[2] Babič M.N., Zupančič J., Gunde-Cimerman N., de Hoog S., Zalar P. (2018). Ecology of the Human Opportunistic Black Yeast Exophiala Dermatitidis Indicates Preference for Human-Made Habitats. Mycopathologia.

[3] de Jong C.C.M., Slabbers L., Engel T.G.P., Yntema J.B., van Westreenen M., Croughs P.D., Roeleveld N., Brimicombe R., Verweij P.E., Meis J.F. (2020). Clinical Relevance of *Scedosporium* Spp. and *Exophiala Dermatitidis* in Patients with Cystic Fibrosis: A Nationwide Study. Med. Mycol..

[4] Chalmers J.D., Chang A.B., Chotirmall S.H., Dhar R., McShane P.J. (2018). Bronchiectasis. Nat. Rev. Dis. Prim..

[5] Polverino E., Goeminne P.C., McDonnell M.J., Aliberti S., Marshall S.E., Loebinger M.R., Murris M., Cantón R., Torres A., Dimakou K. (2017). European Respiratory Society Guidelines for the Management of Adult Bronchiectasis. Eur. Respir. J..

[6] Richardson H., Dicker A.J., Barclay H., Chalmers J.D. (2019). The Microbiome in Bronchiectasis. Eur. Respir. Rev..

[7] Usuda D., Higashikawa T., Hotchi Y., Usami K., Shimozawa S., Tokunaga S., Osugi I., Katou R., Ito S., Yoshizawa T. (2021). Exophiala Dermatitidis. World J. Clin. Cases.

[8] Revankar S.G., Sutton D.A. (2010). Melanized Fungi in Human Disease. Clin. Microbiol. Rev..

[9] Miyoshi S., Tanabe M., Semba M., Sato C., Aoyama S., Watanabe A., Ito R., Hamada K., Watanabe A., Abe M. (2023). Exophiala Dermatitidis Coinfection with Nontuberculous Mycobacteria: A Case Report and Literature Review. Respirol. Case Rep..

[10] Horré R., Schaal K.P., Siekmeier R., Sterzik B., de Hoog G.S., Schnitzler N. (2004). Isolation of Fungi, Especially Exophiala Dermatitidis, in Patients Suffering from Cystic Fibrosis. A Prospective Study. Respiration..

[11] Haase G., Skopnik H., Groten T., Kusenbach G., Posselt H.G. (1991). Long-Term Fungal Cultures from Sputum of Patients with Cystic Fibrosis. Mycoses.

[12] Diemert D., Kunimoto D., Sand C., Rennie R. (2001). Sputum Isolation of Wangiella Dermatitidis in Patients with Cystic Fibrosis. Scand. J. Infect. Dis..

[13] Matsumoto T., Matsuda T., McGinnis M.R., Ajello L. (1993). Clinical and Mycological Spectra of Wangiella Dermatitidis Infections. Mycoses.

[14] Chalmers J.D., Ringshausen F.C., Harris B., Elborn J.S., Posthumus A., Haworth C.S., Pilkington N., Polverino E., Ruddy T., Aliberti S. (2018). Cross-Infection Risk in Patients with Bronchiectasis: A Position Statement from the European Bronchiectasis Network (EMBARC), EMBARC/ELF Patient Advisory Group and European Reference Network (ERN-Lung) Bronchiectasis Network. Eur. Respir. J..

[15] Chalmers J.D., Mall M.A., McShane P.J., Nielsen K.G., Shteinberg M., Sullivan S.D., Chotirmall S.H. (2024). A Systematic Literature Review of the Clinical and Socioeconomic Burden of Bronchiectasis. Eur. Respir. Rev..

[16] Kirchhoff L., Olsowski M., Rath P.-M., Steinmann J. (2019). Exophiala Dermatitidis: Key Issues of an Opportunistic Fungal Pathogen. Virulence.

[17] Boral H., Metin B., Döğen A., Seyedmousavi S., Ilkit M. (2018). Overview of Selected Virulence Attributes in Aspergillus Fumigatus, Candida Albicans, Cryptococcus Neoformans, Trichophyton Rubrum, and Exophiala Dermatitidis. Fungal Genet. Biol..

[18] Suzuki K., Nakamura A., Fujieda A., Nakase K., Katayama N. (2012). Pulmonary Infection Caused by Exophiala Dermatitidis in a Patient with Multiple Myeloma: A Case Report and a Review of the Literature. Med. Mycol. Case Rep..

[19] Myoken Y., Sugata T., Fujita Y., Kyo T., Fujihara M., Katsu M., Mikami Y. (2003). Successful Treatment of Invasive Stomatitis Due to Exophiala Dermatitidis in a Patient with Acute Myeloid Leukemia. J. Oral Pathol. Med. Off. Publ. Int. Assoc. Oral Pathol. Am. Acad. Oral Pathol..

[20] Silva W.C., Gonçalves S.S., Santos D.W.C.L., Padovan A.C.B., Bizerra F.C., Melo A.S.A. (2017). Species Diversity, Antifungal Susceptibility and Phenotypic and Genotypic Characterisation of Exophiala Spp. Infecting Patients in Different Medical Centres in Brazil. Mycoses.

[21] Yoshinouchi T., Yamamoto K., Migita M., Yokoyama T., Nakamura T., Matsuoka M. (2023). Diagnosis and Clinical Management of Exophiala Dermatitidis Pneumonia in a Patient with Anorexia Nervosa: A Case Report. Med. Mycol. Case Rep..

[22] Setoguchi D., Iwanaga N., Ito Y., Ashizawa N., Hirayama T., Takeda K., Ide S., Takemoto S., Tashiro M., Hosogaya N. (2023). Pulmonary Phaeohyphomycosis Due to Exophiala Dermatitidis in a Patient with Pulmonary Non-Tuberculous Mycobacterial Infection. J. Infect. Chemother. Off. J. Japan Soc. Chemother..

[23] Watanabe Y., Sano H., Konno S., Kamioka Y., Hariu M., Takano K., Yamada M., Seki M. (2022). Sinobronchial Syndrome Patients with Suspected Non-Tuberculous Mycobacterium Infection Exacerbated by Exophiala Dermatitidis Infection. Infect. Drug Resist..

[24] Li Z., Tang J., Zhu J., Xie M., Huang S., Li S., Zhan Y., Zeng W., Xu T., Ye F. (2022). The Convoluted Process of Diagnosing Pulmonary Mycosis Caused by Exophiala Dermatitidis: A Case Report. BMC Infect. Dis..

[25] Sekiguchi R., Urabe N., Sakamoto S., Sasaki M., Homma S., Kishi K. (2021). Exophiala Dermatitidis Pneumonia with Bronchiectasis Required Prolonged Voriconazole Treatment. Respirol. Case Rep..

[26] Mukai Y., Nureki S., Hata M., Shigenaga T., Tokimatsu I., Miyazaki E., Kadota J., Yarita K., Kamei K. (2014). Exophiala Dermatitidis Pneumonia Successfully Treated with Long-Term Itraconazole Therapy. J. Infect. Chemother. Off. J. Japan Soc. Chemother..

[27] Bulloch M.N. (2011). The Treatment of Pulmonary Wangiella Dermatitidis Infection with Oral Voriconazole. J. Clin. Pharm. Ther..

[28] Ozawa Y., Suda T., Kaida Y., Kato M., Hasegaw H., Fujii M., Ida M., Nogimura H., Nagayama M., Chida K. (2007). A case of bronchial infection of Wangiella dermatitidis. Nihon Kokyuki Gakkai Zasshi.

[29] Taj-Aldeen S.J., El Shafie S., Alsoub H., Eldeeb Y., de Hoog G.S. (2006). Isolation of Exophiala Dermatitidis from Endotracheal Aspirate of a Cancer Patient. Mycoses.

[30] Mukaino T., Koga T., Oshita Y., Narita Y., Obata S., Aizawa H. (2006). Exophiala Dermatitidis Infection in Non-Cystic Fibrosis Bronchiectasis. Respir. Med..

[31] Barenfanger J., Ramirez F., Tewari R.P., Eagleton L. (1989). Pulmonary Phaeohyphomycosis in a Patient with Hemoptysis. Chest.

[32] Poyntner C., Mirastschijski U., Sterflinger K., Tafer H. (2018). Transcriptome Study of an Exophiala Dermatitidis PKS1 Mutant on an Ex Vivo Skin Model: Is Melanin Important for Infection?. Front. Microbiol..

[33] Poyntner C., Blasi B., Arcalis E., Mirastschijski U., Sterflinger K., Tafer H. (2016). The Transcriptome of Exophiala Dermatitidis during Ex-Vivo Skin Model Infection. Front. Cell. Infect. Microbiol..

[34] Yazdanparast S.A., Mohseni S., De Hoog G.S., Aslani N., Sadeh A., Badali H. (2017). Consistent High Prevalence of Exophiala Dermatitidis, a Neurotropic Opportunist, on Railway Sleepers. J. Mycol. Med..

[35] Lang R., Minion J., Skinner S., Wong A. (2018). Disseminated Exophiala Dermatitidis Causing Septic Arthritis and Osteomyelitis. BMC Infect. Dis..

[36] Kenney R.T., Kwon-Chung K.J., Waytes A.T., Melnick D.A., Pass H.I., Merino M.J., Gallin J.I. (1992). Successful Treatment of Systemic Exophiala dermatitidis Infection in a Patient with Chronic Granulomatous Disease. Clin. Infect. Dis..

[37] Tanamachi C., Hashimoto K., Nakata K., Sagawa K. (2008). A case of pulmonary chromomycosis caused by Exophiala dermatitidis. J. Jpn. Soc. Clin. Microbiol..

[38] Shintani R., Hagiwara E., Yamakawa H., Ikeda S., Kitamura H., Baba T. (2017). Pulmonary phaeohyphomycosis caused by *Exophiala dermatitidis*. JJA Inf..

[39] Goto Y., Murakami N., Yamasaki Y. (2020). Sequelae of pulmonary chromoblastomycosis caused by the viscous species Exophiala dermatitidis in a patient with nontuberculosis mycobacterial disease. Igakukensa.

[40] Masuo M., Hanazawa S., Nukui Y. (2021). Pulmonary chromomycosis caused by *Exophiala dermatitidis* in a patient with pulmonary non-tuberculous mycobacteriosis. J. Jap. Soc. Respir. Endos..

